# Bioavailability of Polycyclic Aromatic Hydrocarbons and their Potential Application in Eco-risk Assessment and Source Apportionment in Urban River Sediment

**DOI:** 10.1038/srep23134

**Published:** 2016-03-15

**Authors:** Xunan Yang, Liuqian Yu, Zefang Chen, Meiying Xu

**Affiliations:** 1 Guangdong Provincial Key Laboratory of Microbial Culture Collection and Application, Guangdong Institute of Microbiology, Guangzhou, China; 2 State Key Laboratory of Applied Microbiology Southern China, Guangzhou, China; 3 Department of Oceanography, Dalhousie University, Halifax, Nova Scotia, Canada

## Abstract

Traditional risk assessment and source apportionment of sediments based on bulk polycyclic aromatic hydrocarbons (PAHs) can introduce biases due to unknown aging effects in various sediments. We used a mild solvent (hydroxypropyl-β-cyclodextrin) to extract the bioavailable fraction of PAHs (a-PAHs) from sediment samples collected in Pearl River, southern China. We investigated the potential application of this technique for ecological risk assessments and source apportionment. We found that the distribution of PAHs was associated with human activities and that the a-PAHs accounted for a wide range (4.7%–21.2%) of total-PAHs (t-PAHs), and high risk sites were associated with lower t-PAHs but higher a-PAHs. The correlation between a-PAHs and the sediment toxicity assessed using tubificid worms (*r* = −0.654, *P* = 0.021) was greater than that from t-PAH-based risk assessment (*r* = −0.230, *P* = 0.472). Moreover, the insignificant correlation between a-PAH content and mPEC-Q of low molecular weight PAHs implied the potiential bias of t-PAH-based risk assessment. The source apportionment from mild extracted fractions was consistent across different indicators and was in accordance with typical pollution sources. Our results suggested that mild extraction-based approaches reduce the potential error from aging effects because the mild extracted PAHs provide a more direct indicator of bioavailability and fresher fractions in sediments.

During the process of urban development, rivers are often physically modified for navigation, minimizing flooding^1^, and even to receive discharge from pipes and gully/urban drainage network. As a result, urban rivers suffer from an abundance of contaminants from anthropogenic activities, such as shipping, road dust, domestic and industrial discharge^2^. Most contaminants accumulate in sediments where their distribution is affected by various physical processes, including the mechanical disturbance at the sediment–water interface resulting from advection and diffusion, particle settling and resuspension, bioturbation, and burial^3^. Numbers of contaminated hotspots have appeared along the river^4^. Docks and canals represent the main hotspots, which commonly receive high levels of organic matter discharged from combined sewage overflow, and contaminants derived from boat traffic^5^.

Polycyclic aromatic hydrocarbons (PAHs) are ubiquitous organic pollutants persisting in urban river sediments^6^,^7^,^8^,^9^. Anthropogenic inputs, such as oil spills, ship traffic, urban runoff, and emission from combustion and industrial processes, are the main sources of PAHs^10^,^11^,^12^,^13^. The elevated concentrations of PAHs, together with their ecological toxicity and health risk for humans, have spawned numerous studies into controlling and removing them^14^,^15^,^16^. However, a large proportion of the total contaminants present are not bioavailable to organisms, and the bioavailability declines as PAHs persist, or age, within a heterogeneous sediment matrix^17^. Therefore, assessment based on bioavailability is considered to be a valuable tool in risk-based approach for remediation or management of contaminated sites^18^,^19^.

Numerous approaches have been developed for assessing the risk of PAHs. Among these, the effects-based, sediment quality guidelines (SQGs) have been implemented worldwide for more than 20 years^14^,^20^,^21^, and are extensively applied in predicting sediment quality based on the toxicity of living organisms^22^. However, doubts have been cast on the applicability of total dose-based approaches, including the SQGs, in different sediment matrixes because the bioaccessibility of PAHs depends on the sediment properties and the aging effects^18^. Hence, biases might occur when the total dose-based approach is used in different sediment matrixes, and some governments have tried to build their local SQGs^23^,^24^. A variety of studies have been conducted on the relationship between bioaccumulation and extraction of non-ionic organic compounds, such as predicting the bioaccumulation using C18-/octadecyl- modified silica^25^,^26^,^27^, mild solvents^26^,^28^, and mixed-solvents^26^,^28^,^29^. Biomimetic extraction technologies were recently developed to predict the bioaccessibility of PAHs in sediment environments^30^. Studies have suggested that the fractions extracted from some mild solvents (e.g., n-butanol, methanol, Tenax, and hydroxypropyl-β-cyclodextrin) were equal to the effect dose of bioaccumulation and biodegradation^30^,^31^,^32^,^33^. Therefore, mild extraction of PAHs may be a suitable new approach for risk assessment of bioavailable PAHs. On the other hand, benthic organisms have been universally used in biomonitoring assays to reflect the organic pollution in aquatic sediments^34^,^35^. Tubificid (such as *Tubifex* sp. and *Limnodrilus* sp.) are the oligochaetes use in heavily contaminated sediment, because they frequently dominate the macrobiotic community in freshwater habitats and generally tolerant to organic pollutants^36^,^37^,^38^,^39^. These worms have developed antioxidant defense mechanisms to prevent cellular damage from reactive oxygen species when exposed to organic pollutants^40^. Glutathione S-transferases (GSTs) are a superfamily of multifunctional enzymes involved in the antioxidant defenses (phase II metabolism), whose activities have been widely used as a biomarker for predicting the toxicity level of organic pollution, including PAHs^40^,^41^,^42^,^43^. Therefore, the relationship between GST activity and mild-extracted PAHs might provide insight into the potential use of mild-extracted PAHs in risk assessment.

Identifying the possible sources and contributions for PAHs in sediment has been proposed in environmental management worldwide^44^,^45^,^46^. Several useful methods have been developed to identify the possible PAH sources in sediments^47^, such as ratios of different PAHs and receptor models. Generally, these methods assume that the compositions of source emissions are constant over the period of ambient and source sampling. However, in reality, the PAHs do not arrive at the receptor during the same period and hence exhibit different bioavailabilities because of variable aging. The fraction with higher bioavailability (less aged) is likely to be of greater interest to environmental managers.

The aim of this study is to reveal the potential of mild extracted PAHs for risk assessment and source apportionment of urban river sediments for future use by regulators. We therefore compare the risk assessments based on total dose with those from the mild extracted fraction of PAHs, and apply the mild extracted fraction to PAHs source apportionment in the urban river sediments.

## Materials and Methods

### Sampling

**Figure 1:**
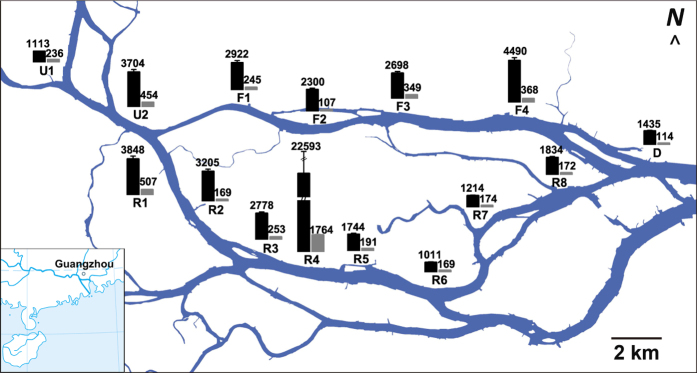
Sampling sites and the contents of PAHs (ng g^−1^ dry sediment) in sediment of the Pearl River, China. The black and grey bars indicate the concentrations of the sum of 16 PAHs and their hydroxypropyl-β-cyclodextrin extractable fraction, respectively. Sites F1–F4 and R1–R8 were located within the front and rear channels, respectively. Sites U1–U2 and D were located at the up and down confluences of the front and rear channels, respectively. The map was created and edited using ArcGIS (version 9.3, ESRI, USA, http://www.esri.com/software/arcgis/) and Origin (OriginLab, Northampton, MA).

Fifteen sample sites were selected in the Guangzhou Section of the Pearl River, China (Fig. 1). Four of these (F1–F4) were located within the front channel, which is a heavily engineered waterway used for sightseeing and water-bus traffic. Eight sites (R1–R8) were located within the rear channel, which serves as freight upstream and industrial buildings are located along the banks. Sites U1–U2 and D were located at the up and down confluences of the front and rear channels, respectively. The surroundings of the sample sites are described in Supplementary Table S1.

Grab samples of surface sediments (triplicate) were collected, freeze-dried (Freezone 4.5, Labconco, USA) and sieved through a 2 mm sieve to remove large debris. To analyze the total content of PAHs, 5 g of sediment was then ground through a 0.15 mm sieve and Soxhlet-extracted with dichloromethane for 24 h. Activated copper was added for desulfurization. The extracts were concentrated and solvent-exchanged to hexane. Each hexane extract was subject to a silica gel-alumina (2:1) glass column (30 cm) for cleanup and fractionation. The column was eluted with 15 mL of hexane to remove the aliphatic hydrocarbons, and the second fraction containing PAHs was eluted with 70 mL of dichloromethane-hexane mixture (3:7). The PAH fraction was concentrated to 2 mL under the gentle N_2_ stream. To analyze the mild extracted fraction (accessible/available fraction, a-PAHs), 1.5 g of sediment were extracted with 30 mL of hydroxypropyl-β-cyclodextrin (HPCD, 50 mM) for 12 h under 150 rpm horizontal vibration. After centrifuging, the HPCD extracts underwent liquid–liquid extraction with hexane. The cleanup and fractionation steps were as described above, and then the extracts were concentrated to 0.2 mL. A known quantity of the recovery standard, m-diphenylbenzene, was added to the sample prior to instrumental analysis.

### Chemical analyses

The 16 EPA priority PAHs are naphthalene, acenaphthene, acenaphthylene, fluorene, phenanthrene, anthracene, fluoranthene, pyrene, benz[a]anthracene, chrysene, benzo[b]fluoranthene, benzo[k]fluoranthene, benzo[a]pyrene, indeno[1,2,3-cd]pyrene, dibenz[a,h]anthracene, and benzo[ghi]perylene. They were analyzed using a gas chromatograph/mass spectrometer detector (Agilent 7890A/5975C, GC/MS, USA). The separation was carried out on a 30 m × 0.25 mm DB-5MS (film thickness 0.25 μm) fused-silica capillary column. The injection and detector port temperatures were 250 °C and 280 °C, respectively. The column temperature was firstly ramped from 35 to 150 °C at 30 °C min^−1^, then was increased to 250 °C at 10 °C min^−1^ and held for 15 min, and lastly increased to 270 °C at 10 °C min^−1^ and held for 8 min. Mass spectra were acquired at the electron ionization mode with an electron multiplier voltage of 1600 eV. The mass scanning ranged between m/z 50 and 500. Data acquisition and processing were controlled by Agilent ChemStation data system. Surrogate standards (perdeuterated PAH compounds) were added to each sample prior to sediment extraction. Surrogate recoveries in Soxhlet-extraction (HPCD-extraction) are as follows: acenaphthene-*d*_10_, 81.1% ± 14.2% (65.7% ± 9.8%); phenanthrene-*d*_10_, 97.3% ± 12.0% (98.7% ± 9.5%); chrysene-*d*_12_, 92.1% ± 8.6% (90.1% ± 8.3%); and perylene-*d*_12_, 90.0% ± 12.4% (99.7% ± 9.8%). For each batch of 24 samples, procedural blanks (solvent) were processed and the detectable amounts of target analytes were deducted as background values during data processing. The detection limits of the method are 0.03–0.63 and 0.35–0.69 ng g^−1^ for bulk PAH and HPCD-extracted PAH, respectively. The reported results were surrogate corrected.

Total organic carbon content (TOC) was determined using the potassium dichromate dilution heat colorimetric method. Grain sizes of sediments were determined by Mastersizer (Malvern 3000, UK).

### Bioassay with tubificid worms

The tubificid worms, *Limnodrilus hoffmeisteri* (Oligochaeta Tubificidae) were maintained and acclimatized under laboratory conditions. They were washed with distilled water prior to analysis. For analysis, the worms were kept in a baker with sediment and river water for 14 days in the dark. A glass bead was used as a control. At the end of the exposure period, the survival of the *L. hoffmeisteri* was evaluated, and the worms were washed and immediately deep frozen. The tissues were homogenized at 4 °C using 0.5 g wet weight *L. hoffmeisteri* per 1 ml phosphate buffer PBS (pH 7.2). The homogenate was centrifuged at 4 °C at 2,500 × g, and the supernatant was used to analyze the GST activities. The GST activity in the *L. hoffmeisteri* extract was evaluated as the formation of a conjugate between glutathione and 1-chloro-2,4-dinitrobenzene at 340 nm in 50 mM potassium phosphate buffer, pH of 6.5 with 1 mM EDTA^48^. The enzyme activity is reported as the number of micromoles of conjugate formed per minute per mg protein. Protein concentrations were estimated using the Coomassie brilliant blue method. Unfortunately, due to an accident, the experiment failed in samples from sites F1 and R1.

### Risk assessment with sediment quality guidelines

The consensus-based SQG approaches were used in this study. The consensus-based probable effect concentration (PEC) value represents a concentration above which adverse effects to benthic organisms are likely. The incidence of sediment toxicity could be evaluated with the ranges of mean PEC quotient (mPEC-Q)^49^,^50^. As established in the SQGs, the PECs are concentrations of individual chemicals above which adverse effects in sediments are expected to frequently occur. Nine PAHs (naphthalene, fluorene, phenanthrene, anthracene, fluoranthene, pyrene, benz[a]anthracene, chrysene, benzo[a]pyrene) were evaluated using the SQGs, according to MacDonald *et al*.^22^. The threshold values of consensus-based PECs^22^,^50^ are listed in Supplementary Table S2.

For each chemical in each sample in the database, a PEC quotient (PEC-Q) was calculated by dividing the concentration of that chemical by the PEC for that chemical^50^:


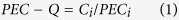


where *C*_*i*_ is the concentration (ng g^−1^) of the chemical *i*, and *PEC*_*i*_ is the threshold effect concentration (ng g^−1^) for the chemical *i* listed in Supplementary Table S2. In particular, the total PAHs (denoted as Σ_9_PAHs) served as an individual chemical when predicting the total effect of PAHs.

For each sample, a mPEC-Q was calculated by dividing the sum of individual quotient for each chemical by the number of PECs evaluated:


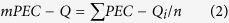


where *PEC-Q*_*i*_ is the PEC quotient of the chemical *i* calculated from Equation (1), and *n* is the number of chemicals selected for calculation. Specifically, the mPEC-Qs of low molecular weight (LMW) PAHs were calculated by evaluating the 2 and 3-ring PAHs (i.e., naphthalene, fluorene, phenanthrene, and anthracene), whereas the mPEC-Qs of high molecular weight (HMW) PAHs were calculated based on the 4- to 6-ring PAHs (i.e., fluoranthene, pyrene, benz[a]anthracene, chrysene, and benzo[a]pyrene).

### Source apportionment

Ratios of specific PAH compounds, such as fluoranthene to the sum of fluoranthene and pyrene (FLT/(FLT + PYR)), benz[a]anthracene to the sum of benz[a]anthracene and chrysene (BaA/(BaA + CHR)), and indeno[1,2,3-cd]pyrene to the sum of indeno[1,2,3-cd]pyrene and benzo[ghi]perylene (IPY/(IPY + BPE)), were calculated to evaluate the possible sources of PAH in sediments. The chemical mass balance (CMB) model, which uses the patterns of special emissions from major source categories to determine the contributions of those sources to a given sample, has been widely used to estimate the source contributions to PAH pollution^51^,^52^. The EPA CMB8.2 modeling software was used in this study. Eight source profiles from the current study in Pearl River Delta^53^ were considered in the CMB8.2 model as follows: coal power plant (CP), coal-fired boiler (CB), coke oven (CO), residential (Re), diesel engines (DE), Gasoline engines (GE), traffic tunnel (TT), and biomass burning (BB).

## Results and Discussion

### Distribution of PAHs and their bioavailability in Pearl River sediment

The Pearl River supports the development of Guangzhou city. Its main channel and waterways are responsible for shipping, flood discharge, and receiving pollution. Figure 1 shows that high total PAHs (Σt-PAHs) were observed in the sediments that were subjected to frequent human activities, such as the water traffic artery (F1–F3), ship building and repairing industries (F4, R1–R5) and areas with riverside human communities (R1–R4, R8). As a result of their high hydrophobicity and persistence, PAHs entering the aquatic ecosystem tend to rapidly adsorb onto suspended particles and settle to the sediment where they become accumulated^54^. Similar to other studies^55^,^56^, we found the highly hydrophobic PAHs such as 4-ring and 5-ring PAHs dominated in sediments (31%–49% and 13%–36%, respectively). However, the concentrations of PAHs did not decrease along the flow direction. The sites subject to frequent human activities (F1–F4, R1–R5) had a concentration of Σt-PAHs around two times higher than sites located in the upstream (U1) and suburban areas (R6 and R7, which is close to orchard land use) (Fig. 1). This confirms that the geographic distribution of PAHs in urban river sediment is associated with hotspots of urban activities.

**Figure 2:**
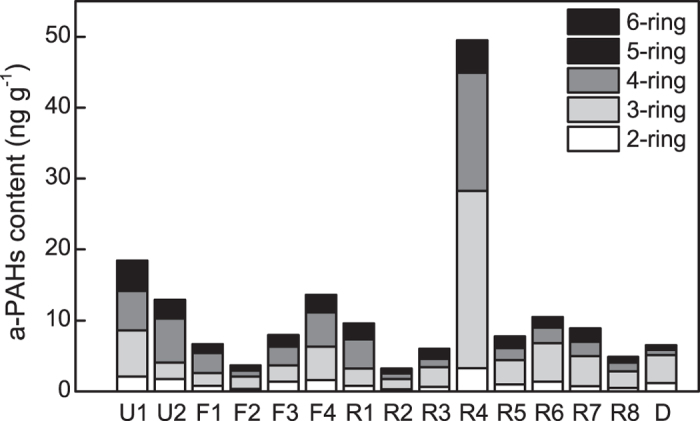
The profiles of a-PAHs at different sites along the Pearl River, China. Sites F1–F4 and R1–R8 were located within the front and rear channels, respectively. Sites U1–U2 and D were located at the up and down confluences of the front and rear channels, respectively.

Although high concentrations of total PAHs have been detected in many river sediments, the presence of PAHs in sediments does not always signify toxicity to the ecosystem^18^. Recent studies suggest that the bioavailable fraction of PAHs is able to account for the eco-toxic effects^57^,^58^ and mild extraction with hydroxypropyl-β-cyclodextrin was demonstrated to be relevant to bioavailability^59^,^60^,^61^. In this study, we extracted the bioavailable PAHs (a-PAHs) from Pearl River sediments with hydroxypropyl-β-cyclodextrin. We found that the dominant a-PAHs were 3-ring and 4-ring PAHs (Fig. 2), which accounted for 4.7%–21.2% of t-PAHs (Supplementary Fig. S1). These percentages were similar to the results from other research^30^. However, the high variability in the percentage of a-PAHs implies that the toxicity of PAHs was not only dependent on t-PAH doses, but might be also controlled by environmental factors. Previous research has suggested that the bioavailability of PAHs in soils and sediments were controlled by the octanol-water partition coefficient (*K*_OW_) of individual PAHs, and the organic compounds content and grain size of sediments^18^,^32^,^62^,^63^. The ratio of a-PAHs to t-PAHs decreased as the lg*K*_OW_ increased (Supplementary Fig. S2). However, significant correlations were observed only between the LMW-PAHs and TOC (*r* = −0.660, *P* < 0.01) and between HMW-PAHs and fine particles (*r* = 0.730, *P* < 0.05) but not for the HMW-PAHs with TOC and LMW-PAHs with fine particles (Supplementary Fig. S3). The reasons might be that sediment organic matter may vary in properties and consist of aggregates of material that may affect the fate of adsorbed PAHs^18^. Furthermore, the PAH distribution relative to organic carbon content might differ between particle-size fractions^64^. The complex interrelation of these physiochemical properties makes it difficult to directly correlate the PAHs concentration with bioavailability even through normalizing to the physiochemical properties (organic carbon) which was conducted in some SQGs^14^. Therefore, sites with relatively low t-PAHs but high a-PAHs (e.g., U1, R6, and R7) could be even more toxic than the heavily polluted sites with high t-PAHs concentration (e.g., F2 and R2). To illustrate this, we compare the a-PAH-based and t-PAH-based eco-risk assessments in the following section.

### Comparison of a-PAHs to traditional eco-risk assessment and bioassay

**Figure 3:**
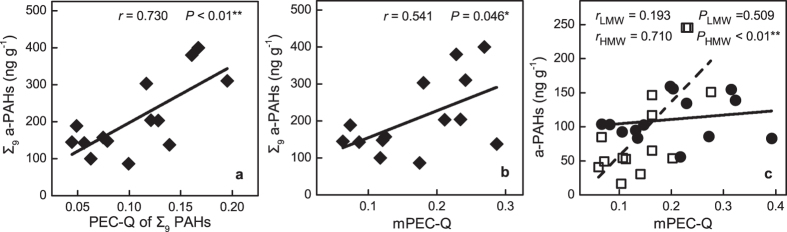
Relationship between a-PAHs and (**a**) PEC-Q of Σ_9_ PAHs, (**b**) mPEC-Q of PAHs and (**c**) mPEC-Q of LMW (solid circle) and HMW PAHs (open square).

Recent studies by Harmsen *et al*.^65^ and Duan *et al*.^18^ suggest that the assessment of PAHs pollution remediation should be based on the bioavailability, which will lead to a realistic appraisal of the potential risks from exposure to contaminants. We compare a-PAH content and consensus-based SQGs in this study. As illustrated in Fig. 3, the correlation between Σ_9_ a-PAHs (hereafter the symbol Σ_9_ represents the sum over the 9 selected PAHs) and PEC-Q of Σ_9_ PAHs was significantly high (*r* = 0.730, *P* < 0.01, Fig. 3a), whereas the correlation between Σ_9_ a-PAHs and mPEC-Q of each individual PAHs was comparatively lower (*r* = 0.541, *P* = 0.048, Fig. 3b). To gain further insights, LMW-PAHs and HMW-PAHs were analyzed separately. As illustrated in Fig. 3c, there is positive correlation between a-PAH content and mPEC-Q of HMW-PAHs but no significant correlation is found between a-PAH content and mPEC-Q of LMW-PAHs. This could explain the discrepancy between a-PAH and t-PAH based approach in this study where LMW-PAHs contributed to 16% to 38% of the total PAHs in sediment samples from different sites (see Fig. 2). The predicted results by SQGs did not correlate well with the bioavailability, possibly due to the regional differences in geochemistry of sediments^14^ and aging effects that might lead to different fate of LMW- and HMW- PAHs^18^ (Supplementary Fig. S3).

**Figure 4:**
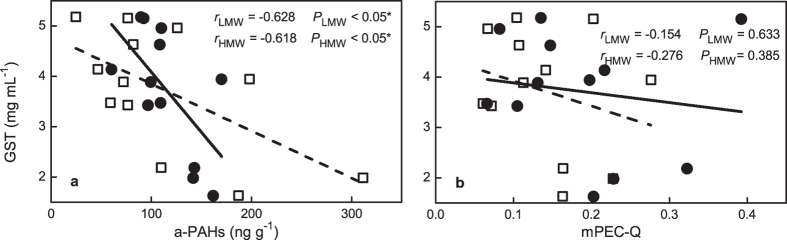
Correlation between GST and (**a**) a-PAHs and (**b**) mPEC-Q. The solid circles and open squares indicate LMW- and HMW- PAHs, respectively.

We next compare the results of sediment toxicity test with *L. hoffmeisteri* with those from the a-PAHs and the mPEC-Q. We selected GST activity as the toxic biomarker because GST plays a crucial role in cellular protection against oxidative stress and toxic compounds such as PAHs^41^,^42^. We found that the GST level of worms in some cases was lower than that in controls (4.69 ± 0.14 mg mL^−1^) (Fig. 4). The depleted GST activities have also been recorded in mussels in the sites polluted with high PAH concentrations^41^. As Akcha *et al*.^41^ suggested, the competition between endogenous substrates and those produced by PAH biotransformation could be responsible for an inhibition of GST. Our results demonstrate a negative correlation between a-PAHs and GST activities in *L. hoffmeisteri* (*r* = −0.654, *P* = 0.021), suggesting that PAHs inhibited the GST activity (either LMW or HMW PAHs, Fig. 4a). However, no significant correlation was observed between GST and t-PAHs (*r* = −0.252, *P* = 0.430) or t-PAHs-based assessments (including the correlations of either mPEC-Q or PEC-Q of the Σ_9_ PAHs, Supplementary Fig. S4). The lack of correlation between t-PAHs and GST activities suggested that the t-PAHs-based method established in northern America might be unable to represent the bioavailability of PAHs in the sediment matrix of this study. Though the eco-toxic tests are generally respond to the status of all contaminants, as for the assessing the GST inhibition by PAHs, however, the a-PAHs has better prediction ability. In all, this suggested the potential applicability of the mild-extraction approach to pollution assessment.

As discussed earlier, the TOC, the percentage of clay, and even other sediment conditions may influence the aging of PAHs and hence affect their bioavailability. The assessment method based on the total dose of PAHs is unable to account for the various sediment properties in different environments. Therefore, we suggest that a-PAHs represent a more suitable and accurate index to determine eco-toxicity.

### The application of mild extraction in source apportionment

**Figure 5:**
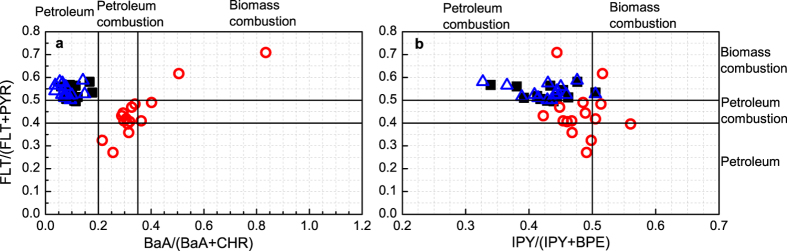
The source apportionment of t-PAHs (black square), a-PAHs (red circle) and r-PAHs (blue triangle) based on molecular ratio diagnostics.

In addition to eco-risk assessment, aging effects should also be considered in source apportionment of PAHs. Most PAH source apportionment in soil or sediment is based on t-PAHs and assumes that the PAHs were mass conservative from the sources to acceptors (i.e., there was no change in source profile between the source and receptor)^51^,^52^. However, PAHs age to different extents after reaching the sediment matrix, where degradation rates are affected by various environmental factors. All of such processes affect the accuracy of the source apportionment. Here, we define the a-PAHs as the fresher (labile or less aged) fraction and the residual PAHs (r-PAHs, the difference between t-PAHs and a-PAHs) as the aged fraction. The source apportionment results for both t-PAHs and r-PAHs differ with types of molecular ratio being used to diagnose (Fig. 5). For example, source based on FLT/(FLT + PYR) suggested that t-PAHs and r-PAHs in sites were primarily determined by biomass combustion, whereas apportionment based on BaA/(BaA + CHR) or IPY/(IPY + BPE) suggested petroleum or petroleum combustion to be the primary source of t-PAHs and r-PAHs. The complex aging and degradation processes led to different fates of PAHs, which made it difficult to appoint the sources of r-PAHs and hence decreased the accuracy of the source apportionment based on the t-PAHs content. These inconsistent source apportionment results imply that further study should be conducted on the accumulation of PAHs in sediments in order to identify the behavior and fate of PAHs. In contrast, the source apportionments based on a-PAHs produced consistent predicting sources under the two pairs of molecular ratios (Fig. 5). This suggests that the a-PAHs obtained through mild extraction, which represents the near-term (or fresher) pollution, might be more meaningful for source apportionments than t-PAHs.

**Figure 6:**
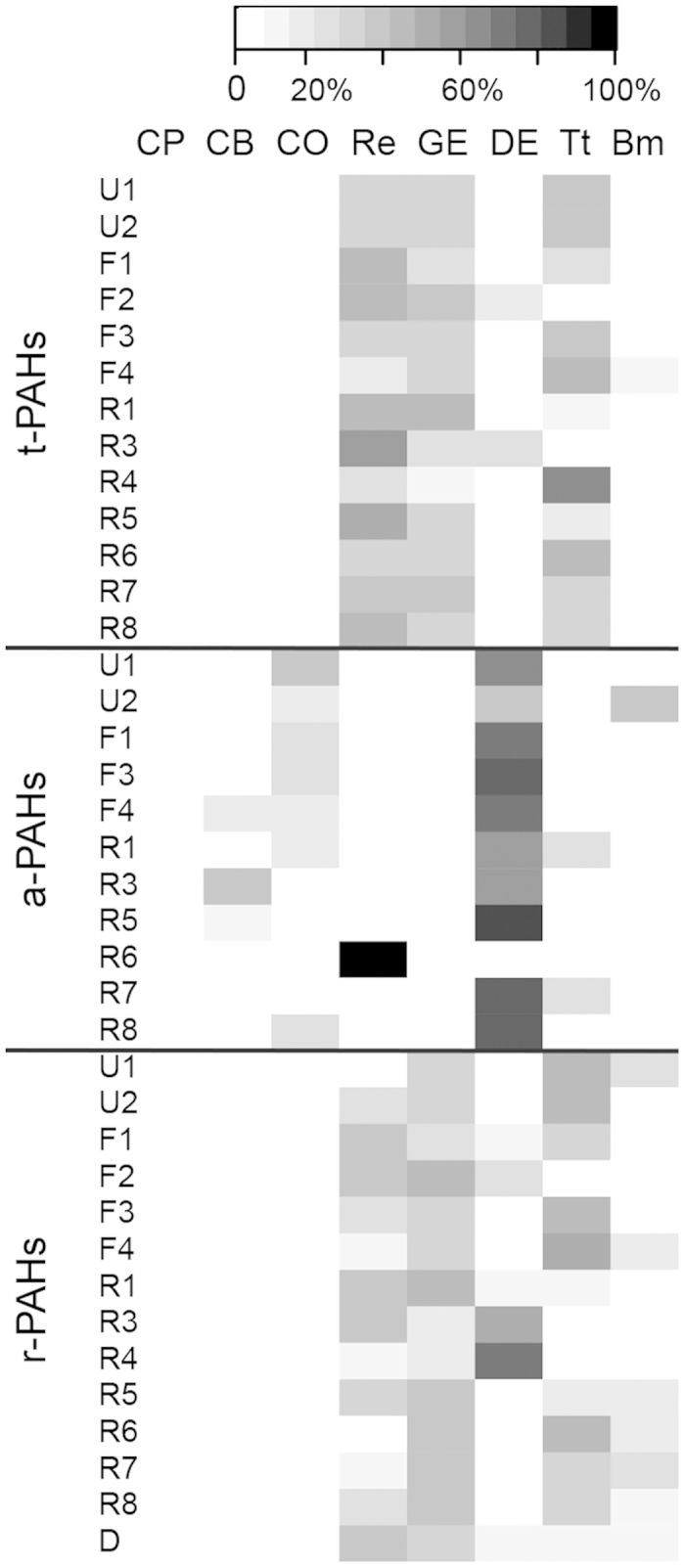
Estimated contributions from sources at sampling sites. The shaded bar represents the percentage of source contribution. The sources were coal power plant (CP), coal-fired boiler (CB), coke oven (CO), residential (Re), diesel engines (DE), Gasoline engines (GE), traffic tunnel (TT), and biomass burning (BB). Sites F1–F4 and R1–R8 located within the front and rear channels, respectively. Sites U1–U2 and D located at the up and down confluences of the front and rear channels, respectively.

The t-PAHs data were decomposed into a-PAHs and r-PAHs, and then each of the three (t-PAHs, a-PAHs, and r-PAHs) was input to the CMB. Using t-PAHs or r-PAHs as input gave similar source contribution profiles; however, those using a-PAHs as input were substantially different (Fig. 6). This implies that the source apportionments of t-PAHs were mainly dependent on the aged fraction, namely the r-PAHs, which is not surprising as in this study r-PAHs accounted for 79% to 96% of t-PAHs in sediment samples. However, the predicted sources of a-PAHs were diesel engines (DE) and coke ovens (CO) (Fig. 6), which were more indicative of the typical PAH pollution sources in urban rivers (dominated by shipping activities and riverside discharges). These results suggest that source apportionment based on a-PAHs provides more accurate prediction than those based on t-PAHs. Furthermore, the a-PAHs represent the least aged PAHs with the highest toxicity potential, and should therefore be of special concern with respect to the current status of contamination.

## Conclusion

In the present work, mild extracted fraction of PAHs showed avantage of predicting the toxicity of PAHs and exhibited higher consistency and rationality in source apportionment than t-PAHs-based approaches. Therefore, for environmental management, we suggest that the mild extracted fraction of PAHs, which was demonstrated to be the bioavailable and un-aged fraction of PAHs, should be taken into account to reduce the error from aging effects. This work suggests that the implementation of mild extracted fraction in risk assessments and source apportionments would be beneficial, though further investigation is still required.

## Additional Information

**How to cite this article**: Yang, X. *et al*. Bioavailability of Polycyclic Aromatic Hydrocarbons and their Potential Application in Eco-risk Assessment and Source Apportionment in Urban River Sediment. *Sci. Rep.* **6**, 23134; doi: 10.1038/srep23134 (2016).

## Supplementary Material

Supplementary Information
