# Supplementary material for: Bioavailability of Polycyclic Aromatic Hydrocarbons and their Potential Application in Eco-risk Assessment and Source Apportionment in Urban River Sediment
Source: Sci Rep. 2016 Mar 15;6:23134. doi: 10.1038/srep23134 (PMC4791542; doi:10.1038/srep23134)
Supplement: Supplementary Information [file srep23134-s1.pdf]

Supplementary information for

Bioavailability of Polycyclic Aromatic Hydrocarbons and their Potential Application in Eco-risk Assessment and Source Apportionment in Urban River Sediment

Xunan Yang[1,2], Liuqian Yu[3], Zefang Chen[1,2], Meiying Xu[1,2,*]

1. Guangdong Provincial Key Laboratory of Microbial Culture Collection and Application, Guangdong Institute of Microbiology, Guangzhou, China; 2. State Key Laboratory of Applied Microbiology Southern China, Guangzhou, China; 3. Department of Oceanography, Dalhousie University, Halifax, Nova Scotia, Canada

*Correspondence and requests for materials should be addressed to M.X. (email: xumy@gdim.cn)

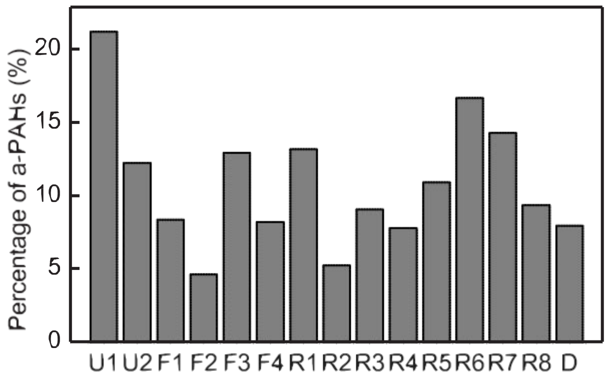

Supplementary Figure S1. The percentages of a-PAHs in t-PAHs at different sites. Sites F1–F4 and R1–R8 were located within the front and rear channels, respectively. Sites U1–U2 and D were located at the up and down confluences of the front and rear channels, respectively.

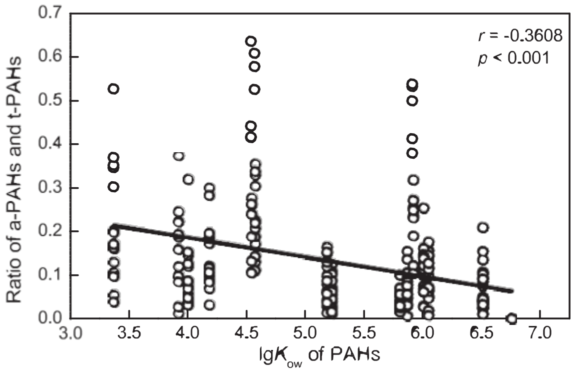

Supplementary Figure S2. The correlation of a-PAHs/t-PAHs and lgK_{ow} of PAHs.

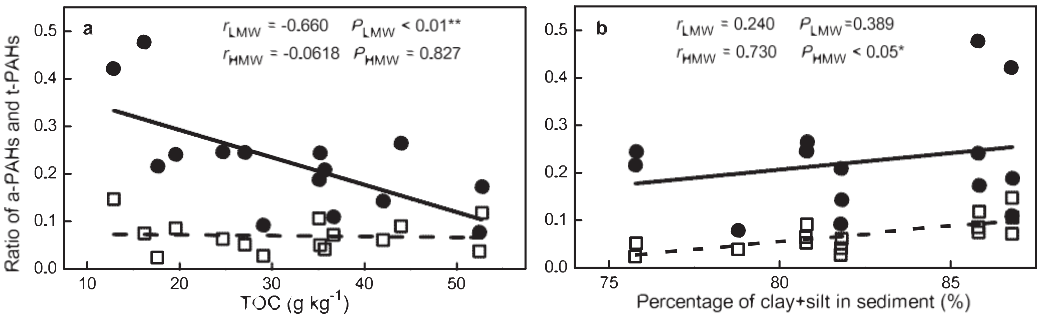

Supplementary Figure S3. The correlation between a-PAHs/t-PAHs and (a) TOC and (b) grain size. The solid circles and open squares indicate LMW- and HMW-PAHs, respectively.

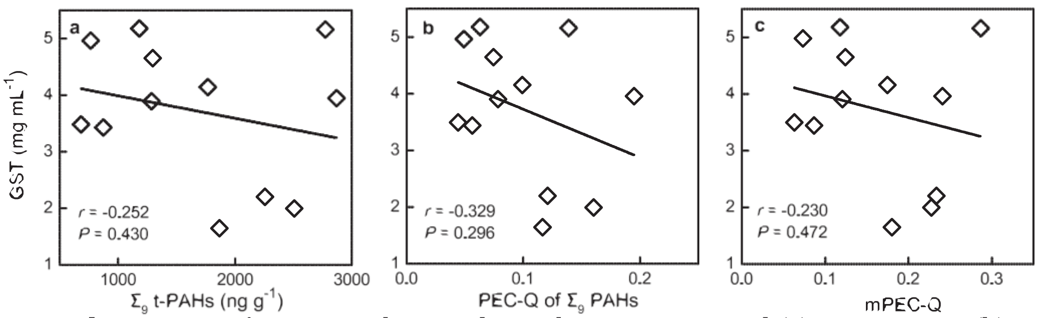

Supplementary Figure S4. The correlation between GST and (a) $\succ_9$ t-PAHs, (b) PEC-Q and (c) mPEC-Q.

Supplementary Table S1 Characteristics of sampling sites

Sites	Longitude and Latitude	Characteristics
U1	26°6′52″N, 113°13′23″E	The debouchment of west channel and Liuxi River, fast flow.
U2	23°6′28″N, 113°13′43″E	The debouchment of rear channel and Huadi river. Huadi river was used to sever the discharge of industrial and domestic wastewater. Face to Huangsha Dock.
F1	23°7′8″N, 113°16′48″E	Beside to Fangzhi Dock, the waterbuses arrive and leav frequently during the day.
F2	23°6′36″N, 113°18′21″ E	Beside to Zhongda Dock, the terminal waterbuses.
F3	23°6′49″N, 113°20′6″E	Beside to Pazhou Dock, built for tourist ship (much less than waterbuses).
F4	23°5′48″N, 113°24′6″E	Beside to Emei alluvion, some constructions in process and wharves on the riverside.
R1	23°5′55″N, 113°14′38″E	Beside to Yongxingjie Dock, surrounded by residential area
R2	23°4′12″N, 113°15′36″E	Beside to the drain outlet of a paper plant. The ship building and repairing industries on the riverside.
R3	23°3′38″N, 113°16′45″E	Several midget plants and large warehouses on the riverside.
R4	23°3′22″N, 113°17′34″E	Beside to Daganwei Wharf and ship repair factory, the outlets of some waterways come from midget industrial zone and residential area.
R5	23°3′18″N, 113°19′12″E	Under the Xingguang Bridge and beside to large cargo berth.
R6	23°2′37″N, 113°20′52″E	In the front of Guanzhou Island, alluvial flat, low flow. Some waders rest on the flat.
R7	23°4′11″N, 113°21′39″E	Beside to the outlet of a waterway from countries and orchards.
R8	23°5′4″N, 113°23′32″E	Beside to a fishermen’s community, lack of sewage discharge network, poor water quality
D	23°5′46″N, 113°24′56″E	The debouchment of the front and rear channel, great water flow.

Supplementary Table S2. Consensus-based SQGs for PAHs in freshwater ecosystems that reflect PECs.

Chemicals	Threshold effect concentrations (ng g^{-1})
naphthalene	561
fluorene	536
phenanthrene	1170
anthracene	845
fluoranthene	2230
pyrene	1520
benz[a]anthracene	1050
chrysene	1290
benzo[a]pyrene	1450
Ì$_9$PAHs	22800
